# Healthcare cost attributable to bronchiolitis: A population-based cohort study

**DOI:** 10.1371/journal.pone.0260809

**Published:** 2021-12-02

**Authors:** Beate Sander, Yaron Finkelstein, Hong Lu, Chenthila Nagamuthu, Erin Graves, Lauren C. Ramsay, Jeffrey C. Kwong, Suzanne Schuh

**Affiliations:** 1 University Health Network, Toronto, Ontario, Canada; 2 University of Toronto, Toronto, Ontario, Canada; 3 ICES, Toronto, Ontario, Canada; 4 Public Health Ontario, Toronto, Ontario, Canada; 5 Department of Paediatrics, The Hospital for Sick Children, Toronto, Ontario, Canada; 6 Research Institute, Division of Child Health Evaluative Sciences, The Hospital for Sick Children, Toronto, Ontario, Canada; PLOS, UNITED KINGDOM

## Abstract

**Objective:**

To determine 1-year attributable healthcare costs of bronchiolitis.

**Methods:**

Using a population-based matched cohort and incidence-based cost analysis approach, we identified infants <12 months old diagnosed in an emergency department (ED) or hospitalized with bronchiolitis between April 1, 2003 and March 31, 2014. We propensity-score matched infants with and without bronchiolitis on sex, age, income quintile, rurality, co-morbidities, gestational weeks, small-for-gestational-age status and pre-index healthcare cost deciles. We calculated mean attributable 1-year costs using a generalized estimating equation model and stratified costs by age, sex, income quintile, rurality, co-morbidities and prematurity.

**Results:**

We identified 58,375 infants with bronchiolitis (mean age 154±95 days, 61.3% males, 4.2% with comorbidities). Total 1-year mean bronchiolitis-attributable costs were $4,313 per patient (95%CI: $4,148–4,477), with $2,847 (95%CI: $2,712–2,982) spent on hospitalizations, $610 (95%CI: $594–627) on physician services, $562 (95%CI: $556–567)] on ED visits, $259 (95%CI: $222–297) on other healthcare costs and $35 ($27–42) on drugs. Attributable bronchiolitis costs were $2,765 (95%CI: $2735–2,794) vs $111 (95%CI: $102–121) in the initial 10 days post index date, $4,695 (95%CI: $4,589–4,800) vs $910 (95%CI: $847–973) in the initial 180 days and $1,158 (95%CI: $1,104–1213) vs $639 (95%CI: $599–679) during days 181–360.

Mean 1-year bronchiolitis costs were higher in infants <3 months old [$5,536 (95%CI: $5,216–5,856)], those with co-morbidities [$17,530 (95%CI: $14,683–20,377)] and with low birthweight [$5,509 (95%CI: $4,927–6,091)].

**Conclusions:**

Compared to no bronchiolitis, bronchiolitis incurs five-time and two-time higher healthcare costs within the initial and subsequent six-months, respectively. Most expenses occur in the initial 10 days and relate to hospitalization.

## Introduction

Bronchiolitis is a viral disease characterized by rhinorrhea, cough and respiratory distress, typically affecting infants under 12 months of age [1]. More than a third of all infants develop bronchiolitis, of whom 3% are admitted to hospital [2]. No drug therapy has proven effective for this disease; bronchiolitis practice guidelines advocate for the use of supportive measures only, and discourage routine use of pharmacotherapy and investigations [1,3–10]. Nonetheless, the use of unnecessary interventions in bronchiolitis is common globally [11–13].

Bronchiolitis carries high morbidity; it represents the leading cause of infant hospitalizations in the Western world, with increasing costs over the past several decades [1,14,15]. In the United States, bronchiolitis-related annual hospitalization expenses exceed $500 million [16,17], with comparable economic burden in other Western countries [18]. Of infants diagnosed with bronchiolitis in the emergency departments (ED), 30–40% require hospitalization [19] and approximately 20% of those discharged home from the ED have unscheduled visits for persistent symptoms within the subsequent 2 weeks [20]. The reported mean annual healthcare utilization for bronchiolitis per 1000 infants under 6 months of age is 17 hospitalizations, 55 ED visits and 132 unplanned office visits [15].

Previous assessments of bronchiolitis management have focused on relatively short-term costs, such as expenditures limited to hospitalizations [15,21], impact of management practice guidelines [22,23], cost-effectiveness of specific treatment interventions [24–26] or on the burden limited to respiratory syncytial virus (a common pathogen in bronchiolitis) [27]. Other researchers have studied patients from a single city or one institution [22,28]. Further, previous work on this topic has focused exclusively on the acute phase of bronchiolitis and did not address the associated economic burden beyond this period, thereby potentially underestimating the full financial impact of this common disease. We are unaware of any studies addressing the overall attributable bronchiolitis-related longer-term healthcare costs in a population from a large region. This information is of importance to advise healthcare professionals and policy-makers on the overall economic impact of bronchiolitis, and to inform implementation of evidence-based best practices in bronchiolitis in order to reduce associated societal and economic burden.

To address this knowledge gap, we conducted a cohort study of infants who presented to an ED or were admitted to a hospital in Ontario, Canada with acute bronchiolitis between April 1, 2003 and March 31, 2014, to determine the total 1-year bronchiolitis-attributable healthcare costs. We hypothesize that the overall healthcare costs among infants who experienced bronchiolitis in the first year of life, are significantly higher than the overall healthcare costs among infants who did not experience bronchiolitis, after matching on sociodemographic and clinical covariates.

## Methods

### Design, setting and subjects

We conducted an incidence-based, propensity score matched cohort study to determine the total attributable healthcare costs in infants with bronchiolitis from the perspective of the healthcare payer (i.e., the Ontario Ministry of Health and Long-term Care).

We identified infants <12 months of age who presented to an ED or were admitted to a hospital in Ontario with acute bronchiolitis (International Classification of Diseases 10^th^ Revision [ICD-10-CA] code J21 or J12.1) between April 1, 2003 and March 31, 2014 (exposed subjects). We assigned the first instance of ED visit/hospitalization for bronchiolitis as the index date. If a physician office visit for bronchiolitis occurred within two days prior to the ED visit/hospitalization, we used the date of the physician office visit as index date. We followed all study subjects for one year after the index date or until death. Residents of Ontario have access to universal public health insurance under the Ontario Health Insurance Plan (OHIP), the single payer for medically necessary services, such as hospital visits and medical procedures [29].

Unexposed subjects (infants without a bronchiolitis diagnosis during the accrual period) were derived from the general Ontario population and matched to exposed subjects (with bronchiolitis) with a 1:1 ratio using propensity scores. Unexposed subjects were randomly assigned an index date based on the distribution of index dates among the exposed infants.

Infants residing outside of Ontario and those without a valid Ontario health card number (Ontario has a universal health plan) were excluded.

The propensity score used to match exposed and unexposed infants was based on the following covariates on index date: sex, age, neighborhood income quintile, rurality, the presence of at least one of thirty-one complex chronic conditions during the first year of life (which include primarily congenital heart disease, chronic lung disease and immune defects) [30], preterm birth status (defined as a birth prior to 37 gestational weeks), small-for-gestational-age status [31] (defined as birth weight under 10^th^ percentile) and pre-index date healthcare cost deciles (measured between the date of birth and 1 day prior to the index visit date). Propensity score matching was conducted with a caliper width equal to 0.2 of the standard deviation [32].

### Data sources

We used population-based health administrative and demographic data applicable to any patient with a valid Ontario health card number, housed at ICES (formerly known as Institute of Clinical Evaluative Sciences). These datasets were linked using unique encoded identifiers and analyzed at ICES. We used the Canadian Institute for Health Information National Ambulatory Care Reporting System (CIHI-NACRS) and Discharge Abstract Database (CIHI-DAD) to obtain comprehensive data for all ED visits and hospitalizations, respectively. The OHIP database was used to identify relevant physician office visits suggestive of bronchiolitis recorded in the two days prior to the index ED visit/hospitalization. The Registered Person’s Database, the central population registry file that enables linkage across population-based health administrative datasets, was used to capture unexposed infants from the general Ontario population, as well as demographic information for all study subjects. Data related to preterm birth and birthweight were captured using the MOMBABY dataset, an ICES-derived cohort that links CIHI-DAD hospitalization records of mothers and their newborns. Health care costs were computed for each infant by obtaining costs associated with use of different types of services (data sources in parentheses): inpatient hospitalization (CIHI-DAD), hospital outpatient clinic, same day surgery, ED visits, visits to dialysis and cancer clinics (CIHI-NACRS), the Ontario Drug Benefit program (ODB), rehabilitation services (National Rehabilitation Reporting System), complex and continuing care (Continuing Care Reporting System), home care services (Home Care Database), OHIP billings, family health network and family health organization (FHO) capitation, the New Drug Funding Program (NDFP), and the Assisted Device Program.

The datasets from this study are held securely in coded form at ICES. While legal data sharing agreements between ICES and data providers (e.g., healthcare organizations and government) prohibit ICES from making the datasets publicly available, access may be granted to those who meet pre-specified criteria for confidential access, available at www.ices.on.ca/DAS (email: das@ices.on.ca). The full dataset creation plan and underlying analytic code are available from the authors upon request, understanding that the computer programs may rely upon coding templates or macros that are unique to ICES and are therefore either inaccessible or may require modification.

This study was approved by the Research Ethics Boards of Sunnybrook Health Sciences Centre and The Hospital for Sick Children, both located in Toronto, Ontario.

### Outcomes

The primary outcome was the mean total publicly funded healthcare cost attributable to bronchiolitis within 1 year of the index ED visit/hospitalization. As secondary outcomes, we examined relevant components of the mean healthcare costs measured in 10-day intervals. Mean total costs of inpatient hospitalizations, ED visits, physician services, drug costs, and other direct healthcare costs were also assessed for the first 30 days, 180 days, and 360 days post index date.

### Analysis

Chi-square tests were used to compare categorical baseline sociodemographic and clinical characteristics between infants with and without bronchiolitis. The ICES patient-level costing algorithm was used to estimate 30-day, 180-day, 360-day, and 1-year healthcare costs (expressed in 2014 Canadian dollars) accrued for each infant. Costs are inflated using the healthcare-specific Consumer Price Index (CPI) reported by Statistics Canada as described in the guidelines on person-level costing [33].

Attributable costs are defined as the costs among the unexposed infants (without bronchiolitis) subtracted from the costs among the exposed infants (with bronchiolitis). We plotted the mean costs among these groups of infants from index date up to 360 days, date of the maximum follow up (March 31, 2015) or death. We also assessed the type of cost–inpatient hospitalizations, ED visits, physician services (in any setting–inpatient, outpatient, office visits), drug costs for outpatients covered by the ODB and NDFP (i.e., primarily children on social assistance), and other costs (all other publicly funded healthcare cost, e.g., laboratory testing)–among exposed and unexposed infants by 1-year, 30-day (split into 10-day categories), 180-day (split into 30-day categories), and 360-day (split into 180-day categories) intervals. Lastly, we examined one-year attributable cost, stratified by age (<3months vs ≥3 months), sex, neighborhood income quintile, rurality (urban vs rural residence), the presence of at least one complex condition during the first year of life [30], preterm birth status, and small-for-gestational-age status, using generalized estimating equation (GEE) modelling. Costs are reported in 2014 Canadian Dollars. All analyses were performed using SAS version 9.4 (SAS Institute, Inc., Cary, NC).

## Results

We identified 58,806 exposed infants hospitalized or diagnosed in the ED with bronchiolitis between April 1, 2003 and March 31, 2014, and matched 58,375 exposed infants to 58,375 unexposed infants (without bronchiolitis). Only 431 (0.7%) exposed infants were excluded due to inability to match an unexposed infant. The mean age of patients was 154 days [SD = 94.6], and patients tended to live in lower-income neighborhoods in urban centers (**Table 1**); 0.1% (n = 35 among exposed, n = 60 among unexposed) of infants died within 1 year of index date, and 4.2% of exposed and 4.0% unexposed infants had at least one chronic condition.

**Table 1:** Selected sociodemographic and clinical characteristics of study infants and their matched counterparts.

Characteristic	Exposed (Bronchiolitis)	Unexposed (No bronchiolitis)	Chi-square
			*P* value*
**Total N**	58,375	58,375	
**Sex**			
**Female**	22,580 (38.7%)	22,460 (38.5%)	0.471
**Male**	35,795 (61.3%)	35,915 (61.5%)	
**Age (days)**			
**Mean ± SD**	154.33 ± 94.64	154.33 ± 94.64	1
**Median (IQR)**	142 (72–224)	142 (72–224)	1
**Rural status**			
**Missing**	183 (0.3%)	156 (0.3%)	< .001
**Urban**	50,659 (86.8%)	51,223 (87.7%)	
**Rural**	7,533 (12.9%)	6,996 (12.0%)	
**Neighbourhood income quintile**			
**Missing**	597 (1.0%)	437 (0.7%)	< .001
**Q1 (lowest)**	15,408 (26.4%)	15,667 (26.8%)	
**Q2**	11,766 (20.2%)	11,732 (20.1%)	
**Q3**	11,371 (19.5%)	11,394 (19.5%)	
**Q4**	10,996 (18.8%)	10,924 (18.7%)	
**Q5 (highest)**	8,237 (14.1%)	8,221 (14.1%)	
**Death in 1st year of life**			
**No**	58,340 (99.9%)	58,315 (99.9%)	0.01
**Yes**	35 (0.1%)	60 (0.1%)	
**No. of days from birth to death in 1st year of life**			
**Mean ± SD**	213.94 ± 95.42	178.77 ± 96.09	0.088
**Median (IQR)**	227 (140–300)	156 (108–261)	0.093
**Total No. of Chronic Conditions**			
**Mean ± SD**	0.06 ± 0.35	0.05 ± 0.31	< .001
**Median (IQR)**	0 (0–0)	0 (0–0)	0.09
Presence of chronic respiratory, cardiac, or immunologic disease***	1,632 (2.8%)	1,417 (2.4%)	< .001
**Preterm Birth**	7,506 (12.9%)	7,745 (13.3%)	0.038
**Low Birthweight**	5,580 (9.6%)	5,790 (9.9%)	0.07

Note: Small cells ≤5 have been suppressed.

*P-values were calculated using chi-square and Kruskal-Wallis tests for categorical and continuous variables, respectively.

**Mental retardation is excluded from this table, as no one in cohort had a ’yes’ for this flag.

**Fig 1:**
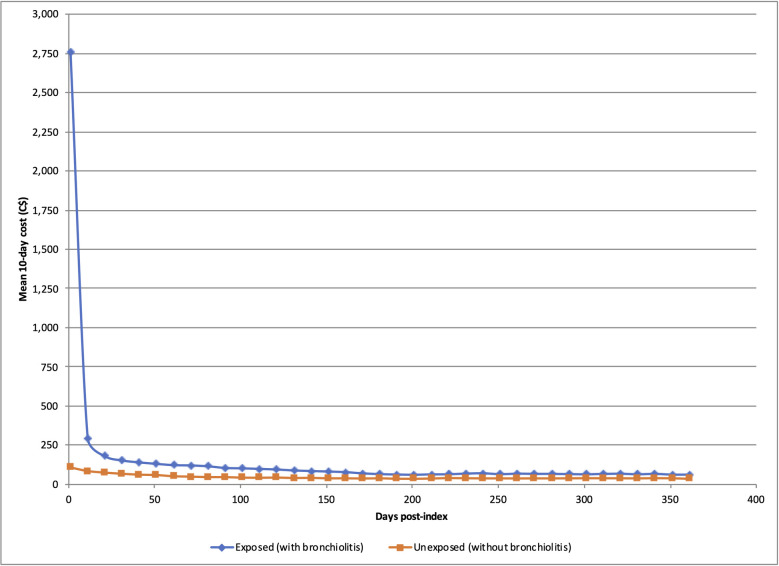
Mean healthcare costs up to 360 days post-index date (in 10-day intervals) among patients who survived 1^st^ year of life.

Exposed infants who survived the first year of life had higher mean healthcare cost within the 360-day period following index date, both overall and across every 10-day interval within this period, compared to unexposed infants (**Fig 1**). Total mean costs attributable to bronchiolitis over a one-year period from index date were $4,313 per subject (95% CI: $4,148-$4,477)—**Table 2**. The greatest excess costs incurred were due to inpatient hospitalizations, followed by physician services, ED visits, other healthcare costs and drug costs. This trend was also observed for most of the initial 30-day, 180-day, and 360-day intervals from index date. Costs attributable to bronchiolitis were seven times higher during the initial 180 days compared to the latter 180 days post-index date (**Table 2**). Healthcare costs in infants with bronchiolitis compared to those without bronchiolitis were five times higher during days 0–180 and almost two-times higher during days 181–360 after index date (**Table 2**).

**Table 2:** Mean attributable costs of bronchiolitis*.

		Exposed (Bronchiolitis) N = 58,340	Unexposed (No bronchiolitis) N = 58,315	
	Type of Cost	Cost ($\bar{x}$, 95% CI)	Cost ($\bar{x}$, 95% CI)	Attributable Cost** ($\bar{x}$, 95% CI)
**One year (365 days)**	Hospitalizations	$3,438 ($3,327, $3,549)	$591 ($512, $669)	$2,847 ($2,712, $2,982)
	ED visits	$702 ($697, $706)	$140 ($138, $142)	$562 ($556, $567)
	Physician services	$1,142 ($1,126, $1,157)	$532 ($524, $539)	$610 ($594, $627)
	Drug costs	$52 ($46, $59)	$18 ($14, $22)	$35 ($27, $42)
	Other costs	$537 ($504, $570)	$278 ($260, $297)	$259 ($222, $297)
	**Total cost**	**$5,871 ($5,731, $6,010)**	**$1,558 ($1,464, $1,651)**	**$4,313 ($4,148, $4,477)**
**Initial 360 day costs**	** **			
0–180 days	Hospitalizations	$3,013 ($2,922, $3,103)	$405 ($350, $461)	$2,607 ($2,503, $2,712)
	ED visits	$546 ($542, $549)	$64 ($63, $66)	$482 ($478, $485)
	Physician services	$800 ($790, $810)	$290 ($285, $296)	$510 ($499, $521)
	Drug costs	$24 ($22, $26)	$8 ($6, $10)	$16 ($13, $19)
	Other costs	$313 ($295, $330)	$142 ($134, $151)	$170 ($151, $189)
	**Total cost**	**$4,695 ($4,589, $4,800)**	**$910 ($847, $973)**	**$3,784 ($3,664, $3,905)**
181–360 days	Hospitalizations	$418 ($376, $459)	$182 ($149, $216)	$235 ($182, $289)
	ED visits	$152 ($150, $154)	$74 ($73, $75)	$78 ($75, $81)
	Physician services	$334 ($326, $343)	$237 ($233, $240)	$98 ($89, $107)
	Drug costs	$28 ($23, $33)	$9 ($7, $11)	$19 ($13, $24)
	Other costs	$227 ($210, $244)	$137 ($126, $148)	$90 ($70, $110)
	**Total cost**	**$1,158 ($1,104, $1,213)**	**$639 ($599, $679)**	**$519 ($452, $586)**

*Costing begins on day of index.

**Attributable cost = (cost of exposed)—(cost of unexposed).

Infants less than 3 months of age, those with at least one complex chronic condition and infants born prematurely had higher attributable costs over the 1-year period post-index date compared to their counterparts without these characteristics (**Table 3**). There were no significant differences in attributable 1-year costs incurred in males versus females, in infants living in rural versus urban areas or in those across neighborhood income quintiles (**Table 3**).

**Table 3:** Mean attributable costs of bronchiolitis over one year (365 days) following index visit (stratified analysis).

	Exposed (Bronchiolitis)		Unexposed (No bronchiolitis)		
	N	Cost ($\bar{x}$, 95% CI)	N	Cost ($\bar{x}$, 95% CI)	Attributable Cost** ($\bar{x}$, 95% CI)
**Age**					
** < 3months**	15,093	$7,449 ($7,196, $7,701)	15,075	$1,910 ($1,701, $2,120)	$5,536 ($5,216, $5,856)
** ≥ 3months**	43,247	$5,320 ($5,154, $5,485)	43,240	$1,435 ($1,332, $1,537)	$3,885 ($3,694, $4,076)
**Sex**					
** Female**	22,559	$6,125 ($5,882, $6,367)	22,434	$1,540 ($1,374, $1,707)	$4,580 ($4,293, $4,868)
** Male**	35,781	$5,710 ($5,543, $5,878)	35,881	$1,568 ($1,458, $1,679)	$4,140 ($3,943, $4,338)
**Neighbourhood income quintile**					
** Missing**	597	$5,795 ($5,005, $6,584)	437	$931 ($704, $1,158)	$4,796 ($4,028, $5,563)
** Q1 (lowest)**	15,397	$6,110 ($5,806, $6,413)	15,644	$1,656 ($1,435, $1,878)	$4,449 ($4,078, $4,819)
** Q2**	11,759	$5,731 ($5,462, $6,000)	11,718	$1,707 ($1,459, $1,955)	$4,016 ($3,657, $4,374)
** Q3**	11,364	$5,959 ($5,636, $6,282)	11,385	$1,583 ($1,397, $1,770)	$4,363 ($4,001, $4,724)
** Q4**	10,988	$5,636 ($5,350, $5,922)	10,915	$1,537 ($1,354, $1,721)	$4,084 ($3,757, $4,411)
** Q5 (highest)**	8,235	$5,819 ($5,428, $6,211)	8,216	$1,181 ($1,070, $1,292)	$4,635 ($4,231, $5,038)
**Rural status**					
** Missing**	183	$5,183 ($4,144, $6,222)	156	$798 ($468, $1,127)	$4,342 ($3,321, $5,364)
** Urban**	50,628	$5,944 ($5,792, $6,095)	51,170	$1,609 ($1,504, $1,714)	$4,332 ($4,151, $4,512)
** Rural**	7,529	$5,397 ($5,049, $5,745)	6,989	$1,200 ($1,067, $1,333)	$4,191 ($3,822, $4,560)
**Presence of any complex chronic condition** ***					
** No**	55,925	$4,920 ($4,825, $5,016)	56,019	$1,197 ($1,125, $1,269)	$3,723 ($3,605, $3,841)
** Yes**	2,415	$27,877 ($25,506, $30,247)	2,296	$10,351 ($8,791, $11,910)	$17,530 ($14,683, $20,377)
**Preterm Birth**					
** No**	50,840	$5,311 ($5,181, $5,441)	50,597	$1,193 ($1,130, $1,256)	$4,110 ($3,969, $4,252)
** Yes**	7,500	$9,664 ($9,046, $10,283)	7,718	$3,947 ($3,376, $4,517)	$5,718 ($4,887, $6,549)

*Costing begins on day of index.

**Attributable cost = (cost of exposed)—(cost of unexposed).

***Presence of at least one of 30 complex chronic conditions during first year of life. Full list of conditions outlined in S2 Table.

## Discussion

This study demonstrates that bronchiolitis incurs five-time and two-time higher healthcare costs within the initial and subsequent six months post-index presentation, respectively, compared to costs in infants without bronchiolitis. While the 10-day costs attributable to bronchiolitis were highest during the initial 10 days post-index date, higher costs persisted at 1-year post-index date. The majority of the bronchiolitis-attributable costs were related to hospitalizations.

Our study identified particularly higher attributable costs of bronchiolitis among infants less than 3 months of age, those with at least one complex chronic condition, and in infants delivered prematurely. These factors have previously been identified as predictors of severe bronchiolitis [34–40]. A recent study from Ontario also found that infants with these characteristics are at a relatively high risk of dire outcomes shortly after ED discharge, including intensive care unit admission or death [41].

Several studies have assessed the economic burden of hospitalizations associated with lower respiratory tract infections, such as bronchiolitis [15,18,42]. A study conducted in Germany considered the societal (costs within and outside the healthcare system) and healthcare-related costs of lower respiratory tract infections in children aged 0–36 months [18] and estimated the direct medical costs associated with bronchiolitis at €3,170 ($4,787 CAD, 2005 exchange rate of €1 = $1.51 [43]) per hospitalized patient [18]. A study of children under two years of age hospitalized for bronchiolitis in the United States reported an average cost per hospitalization of $3,799 USD ($4,293 CAD, 2006 exchange rate of $1 USD = $1.13 CAD [43]) [15]. Another study from the Seattle Children’s Hospital in the United States estimated the total cost per hospitalized bronchiolitis case between $4,446 and $5,742 USD ($4,891 to $6,316 CAD 2014 exchange rate $1 USD = $1.1 CDN, [43]) [42]. These studies were limited by a small number of bronchiolitis cases [18], use of a single-center design [42], and a focus on hospitalizations during a single year almost two decades ago [15]. In contrast, our study examined healthcare costs related to overall bronchiolitis care incurred over a one-year period. Interestingly, we found that our estimate of the bronchiolitis-attributable costs for the initial hospitalization is lower than the previously reported estimates [15,18,42]. While the exploration of reasons for this finding is beyond the scope of this study, plausible factors which may have contributed to this cost disparity may include different length of hospital stay, disparate costs of healthcare services in various jurisdictions, and social factors [44].

This study highlights that hospitalization costs represent the largest contributor to the overall high bronchiolitis-related economic burden. A recent multi-national pediatric emergency research network study found that more than 30% of infants hospitalized for bronchiolitis receive no evidence-based supportive therapies that must be administered in hospital and that hospitalization is impacted by location of care, independent of bronchiolitis severity [12]. Therefore, enhanced best practices of resource utilization need to be implemented to decrease potentially unnecessary hospitalizations and cost of care.

Our study has several limitations. The potential for disease misclassification bias is inherent in studies using large administrative healthcare data. Although the NACRS database includes a diagnostic code for bronchiolitis which enabled us to identify bronchiolitis-related ED visits and hospitalizations, such is not the case for OHIP codes. Therefore, some bronchiolitis-related office visits may have been misclassified. However, we only used OHIP codes to adjust the index date if office visits suggestive of bronchiolitis occurred within two days prior to the ED visit/hospitalization (i.e., we did not identify infants with bronchiolitis using OHIP codes). The impact of potential misclassification is therefore expected to be small.

While we accounted for baseline differences in known co-morbidities, other complex conditions which impact healthcare utilization and costs may not have been diagnosed prior to the index date. Therefore, we do not know whether such unidentified co-morbidities would be distributed equally among patients with and without bronchiolitis. However, we also included healthcare costs prior to the index date as a proxy for healthcare utilization due to co-morbidities and to account for healthcare-seeking behavioral differences of caregivers.

Another limitation is that our study design allowed us to focus only on healthcare costs from a healthcare payer perspective (i.e., publicly funded healthcare cost). In Ontario, drugs are only publicly funded for (but not limited to) hospitalized patients and for outpatients aged ≥65 years or those on social assistance. Therefore, drug costs not publicly funded are not captured in our study. Further, indirect costs from the societal perspective (e.g., workdays lost by caregivers) were not included. Therefore, our cost estimate is conservative.

The strengths of our study include the availability of comprehensive, population-based data, which allowed us to include a broad set of variables in the propensity score, thus enabling us to account for patient-level differences between infants with and without bronchiolitis and to reduce bias. Further, our analysis accounted for all publicly funded healthcare costs and extended beyond the initial ED visit or hospitalization. Therefore, our study provides a comprehensive estimate of longer-term healthcare costs of bronchiolitis. Our findings are based on a large, province-wide, population-based bronchiolitis cohort managed over an 11-year period, with no socioeconomic, geographic or other restrictions, which increases the generalizability of the findings.

In summary, we found that costs for infants with bronchiolitis were five-time and two-time higher compared to infants without bronchiolitis within the initial and subsequent six-month post-index date periods, respectively. The 10-day attributable costs were highest during the initial 10 days, and remained higher one year later. The majority of the bronchiolitis-attributable costs relate to hospitalizations.

## Supporting information

S1 Table Mean attributable costs of bronchiolitis for the initial 30 days and 180 days from index*. (DOCX)

S2 Table List of complex chronic conditions. (DOCX)
