# Supplementary material for: Healthcare cost attributable to bronchiolitis: A population-based cohort study
Source: PLoS One. 2021 Dec 2;16(12):e0260809. doi: 10.1371/journal.pone.0260809 (PMC8639079; doi:10.1371/journal.pone.0260809)
Supplement: S1 Table — (DOCX) [file pone.0260809.s001.docx]

**S1 Table: Mean attributable costs of bronchiolitis for the initial 30 days and 180 days from index***

|  |  | **Exposed (Bronchiolitis)**  **N= 58,340** | **Unexposed (No bronchiolitis)**  **N= 58,315** |  |
| --- | --- | --- | --- | --- |
|  | **Type of Cost** | **Cost (x̄, 95% CI)** | **Cost (x̄, 95% CI)** | **Attributable Cost** (x̄, 95% CI)** |
| **Initial 30 day costs** |  |  |  |  |
| 0-10 days | Hospitalizations | $1,941 ($1,916, $1,967) | $68 ($60, $77) | $1,873 ($1,847, $1,899) |
|  | ED visits | $381 ($378, $383) | $4 ($4, $5) | $376 ($374, $378) |
|  | Physician services | $365 ($361, $368) | $25 ($24, $26) | $339 ($336, $343) |
|  | Drug costs | $3 ($3, $4) | $1 ($0, $1) | $3 ($3, $3) |
|  | Other costs | $75 ($73, $77) | $13 ($12, $14) | $62 ($60, $64) |
|  | **Total cost** | **$2,765 ($2,735, $2,794)** | **$111 ($102, $121)** | **$2,654 ($2,623, $2,684)** |
| 11-20 days | Hospitalizations | $214 ($200, $227) | $49 ($42, $56) | $164 ($149, $180) |
|  | ED visits | $14 ($13, $15) | $4 ($4, $5) | $10 ($9, $11) |
|  | Physician services | $51 ($50, $53) | $23 ($22, $23) | $29 ($27, $31) |
|  | Drug costs | $1 ($1, $1) | $1 ($0, $1) | $1 ($0, $1) |
|  | Other costs | $24 ($22, $25) | $12 ($11, $13) | $12 ($10, $13) |
|  | **Total cost** | **$304 ($288, $319)** | **$88 ($80, $96)** | **$215 ($198, $233)** |
| 21-30 days | Hospitalizations | $115 ($105, $125) | $41 ($34, $47) | $75 ($63, $87) |
|  | ED visits | $14 ($13, $14) | $4 ($4, $4) | $10 ($9, $10) |
|  | Physician services | $38 ($37, $40) | $22 ($21, $22) | $17 ($15, $18) |
|  | Drug costs | $1 ($1, $2) | $0 ($0, $1) | $1 ($1, $1) |
|  | Other costs | $21 ($20, $23) | $12 ($11, $12) | $10 ($8, $11) |
|  | **Total cost** | **$190 ($178, $201)** | **$78 ($71, $86)** | **$111 ($98, $125)** |
| **Initial 180 day costs** |  |  |  |  |
| 0-30 days | Hospitalizations | $2,223 ($2,185, $2,262) | $149 ($130, $168) | $2,074 ($2,032, $2,116) |
|  | ED visits | $406 ($403, $408) | $12 ($11, $12) | $394 ($391, $396) |
|  | Physician services | $444 ($439, $448) | $65 ($63, $67) | $379 ($374, $384) |
|  | Drug costs | $6 ($5, $6) | $1 ($1, $2) | $4 ($4, $5) |
|  | Other costs | $110 ($106, $114) | $30 ($28, $32) | $80 ($76, $84) |
|  | **Total cost** | **$3,188 ($3,144, $3,232)** | **$257 ($236, $278)** | **$2,930 ($2,883, $2,978)** |
| 31-60 days | Hospitalizations | $237 ($215, $259) | $87 ($72, $102) | $150 ($123, $177) |
|  | ED visits | $34 ($33, $35) | $11 ($11, $12) | $23 ($22, $24) |
|  | Physician services | $93 ($90, $96) | $56 ($54, $58) | $37 ($34, $40) |
|  | Drug costs | $3 ($3, $4) | $1 ($1, $2) | $2 ($2, $3) |
|  | Other costs | $50 ($46, $53) | $27 ($26, $29) | $22 ($19, $26) |
|  | **Total cost** | **$417 ($392, $442)** | **$183 ($165, $200)** | **$234 ($204, $265)** |
| 61-90 days | Hospitalizations | $192 ($170, $214) | $57 ($45, $69) | $135 ($110, $160) |
|  | ED visits | $31 ($30, $32) | $11 ($10, $11) | $20 ($19, $22) |
|  | Physician services | $81 ($78, $83) | $48 ($47, $50) | $32 ($30, $35) |
|  | Drug costs | $4 ($3, $4) | $1 ($1, $2) | $2 ($2, $3) |
|  | Other costs | $47 ($44, $50) | $27 ($25, $29) | $20 ($16, $24) |
|  | **Total cost** | **$354 ($330, $379)** | **$144 ($131, $158)** | **$210 ($182, $238)** |
| 91-120 days | Hospitalizations | $155 ($136, $174) | $46 ($35, $56) | $110 ($88, $131) |
|  | ED visits | $29 ($28, $30) | $10 ($10, $11) | $18 ($17, $19) |
|  | Physician services | $72 ($70, $74) | $46 ($44, $47) | $26 ($24, $29) |
|  | Drug costs | $4 ($3, $4) | $2 ($1, $2) | $2 ($2, $3) |
|  | Other costs | $46 ($43, $49) | $26 ($24, $28) | $20 ($16, $24) |
|  | **Total cost** | **$306 ($284, $328)** | **$129 ($118, $141)** | **$176 ($152, $201)** |
| 121-150 days | Hospitalizations | $132 ($113, $150) | $43 ($33, $52) | $89 ($68, $110) |
|  | ED visits | $27 ($26, $28) | $11 ($10, $11) | $16 ($15, $17) |
|  | Physician services | $65 ($63, $67) | $43 ($41, $44) | $22 ($20, $25) |
|  | Drug costs | $4 ($3, $4) | $1 ($1, $2) | $3 ($2, $3) |
|  | Other costs | $45 ($41, $48) | $26 ($24, $27) | $19 ($15, $23) |
|  | **Total cost** | **$272 ($251, $292)** | **$123 ($112, $134)** | **$149 ($126, $172)** |
| 151-180 days | Hospitalizations | $103 ($89, $118) | $34 ($27, $42) | $69 ($53, $86) |
|  | ED visits | $25 ($24, $26) | $11 ($11, $12) | $14 ($13, $15) |
|  | Physician services | $59 ($57, $60) | $40 ($39, $42) | $18 ($16, $20) |
|  | Drug costs | $4 ($3, $5) | $2 ($1, $2) | $3 ($2, $3) |
|  | Other costs | $44 ($40, $47) | $26 ($24, $29) | $17 ($13, $21) |
|  | **Total cost** | **$235 ($218, $251)** | **$114 ($104, $123)** | **$121 ($102, $140)** |

**Costing begins on day of index*

***Attributable cost = (cost of exposed) - (cost of unexposed)*
