# Supplementary material for: Healthcare cost attributable to bronchiolitis: A population-based cohort study
Source: PLoS One. 2021 Dec 2;16(12):e0260809. doi: 10.1371/journal.pone.0260809 (PMC8639079; doi:10.1371/journal.pone.0260809)
Supplement: S2 Table — (DOCX) [file pone.0260809.s002.docx]

| **Chronic condition** |
| --- |
| Amino Acid Metabolism |
| Bone & Joint Anomalies |
| Brain & Spinal Cord Malformation |
| Carbohydrate Metabolism |
| Cardiomyopathies |
| Chromosomal Anomalies |
| Chronic Liver Disease & Cirrhosis |
| Chronic Renal Failure |
| Chronic Respiratory Disease |
| Central Nervous System Degeneration & Disease |
| Conduction Disorders & Dysrhythmias |
| Congenital Anomalies |
| Cystic Fibrosis |
| Diaphragm Abdominal Wall |
| Epilepsy |
| Gastrointestinal Congenital Anomalies |
| Heart and Great Vessel Malformations |
| Hereditary Anemias |
| Hereditary Immunodeficiency |
| Human Immunodeficiency Virus Disease |
| Infantile Cerebral Palsy |
| Inflammatory Bowel Disease |
| Lipid Metabolism |
| Malignancy |
| Muscular Dystrophies and Myopathies |
| Other Congenital Anomalies |
| Other Metabolic Disorders |
| Respiratory Malformations |
| Sickle Cell Disease |
| Storage Disorders |

**S2 Table: List of complex chronic conditions**

List of complex chronic conditions adapted from: Feudtner et al. Pediatric deaths attributable to complex chronic conditions: A population based study of Washington State, 1980-1997; Pediatrics 2000.
